# A multi-fractal vascular tree model for characterizing blood flow in cardiovascular system

**DOI:** 10.1038/s41598-026-44408-5

**Published:** 2026-05-27

**Authors:** Jing Fu, Xiawen Yang, Gaoran Xu, Yiwei Ding, Yuanyuan Li, Maochun Li, Ming Yue, Dengzheng Zhang, Wenlong Xie, Li Li, Juyi Li

**Affiliations:** 1 Department of Pharmacy, Tongji Medical College, The Central Hospital of Wuhan, Huazhong University of Science and Technology, Wuhan, 430014 Hubei China; 2 Key Laboratory for Molecular Diagnosis of Hubei Province, Tongji Medical College, The Central Hospital of Wuhan, Huazhong University of Science and Technology, Wuhan, 430014 Hubei China; 3 Hubei Key Laboratory of Natural Active Polysaccharides, Union Hospital, Tongji Medical College, Huazhong University of Science and Technology, Hubei, Wuhan, 430022 Hubei China; 4 Department of Thyroid and Breast Surgery, Tongji Medical College, The Central Hospital of Wuhan, Huazhong University of Science and Technology, Wuhan, 430014 Hubei China; 5 Department of Pharmacy, Xianning Central Hospital, The First Affiliated Hospital of Hubei University of Science and Technology, Xianning, 437000 Hubei China; 6 Nuclear Medicine and PET-CT/MR Center, Renmin Hospital of Wuhan University, 99 Zhangzhidong Road, Wuchang district, Wuhan, 430060 Hubei China; 7 Yicheng People’s Hospital, Public Health Sector, Xiangyang, 441400 China Hubei

**Keywords:** Cardiovascular system, Blood flow, Fractal vascular tree, Newtonian fluid, Permeability, Cardiology, Computational biology and bioinformatics, Engineering, Mathematics and computing, Physiology

## Abstract

The cardiovascular system’s hemodynamics is pivotal for tissue oxygen and nutrient delivery. However, accurately characterizing its blood flow characteristics remains a major challenge due to the complex distribution and geometry of the cardiovascular system. This study introduces a novel multi - fractal vascular tree (MFVT) model. By integrating heterogeneous fractal dimensions and scaling laws, considering vessel branching and tortuosity, the model depicts blood flow across various vessels. Formulas for blood flow rates in single vessels, fractal vessel trees, and bundles of fractal vessel trees were derived, along with the analytical expression for cardiovascular system permeability. The model’s reliability was verified via four case studies, with a maximum relative error of less than 5% between theoretical and experimental values. Sensitivity analyses showed that parameters significantly influenced blood flow. For instance, as the maximum diameter of zero-level vessels increased from 0.2 to 4 mm, the flow rate rose notably. When the diameter ratio increased from 0.5 to 0.8, the flow rate also increased, especially at higher pressure differences. An increase in the initial length of zero-level vessels from 5 to 50 mm led to a decrease in the flow rate. These phenomena indicate that blood flow resistance is negatively correlated with vessel diameter and positively correlated with vessel length. This work advances the mechanistic understanding of blood flow distribution and provides a computational tool for studying vascular pathologies.

## Introduction

The cardiovascular system, a hierarchically branched network spanning from macroscopic arteries to microscopic capillaries, plays a pivotal role in maintaining hemodynamic homeostasis and tissue perfusion^1^. Hemodynamic parameters, including flow velocity, pressure distribution, and wall shear stress, are intrinsically linked to physiological functions and pathological conditions such as atherosclerosis, hypertension, and vascular remodeling^2^. Hemodynamic metrics such as time-averaged wall shear stress (TAWSS) and oscillatory shear index (OSI) are well-established predictors of vascular tissue degradation, endothelial dysfunction, and aneurysm progression. Regions exhibiting low TAWSS and high OSI undergo markedly accelerated pathological remodeling, reflecting self-reinforcing flow-induced damage mechanisms^3,4^. These associations highlight the importance of incorporating local hemodynamic indices into vascular biomechanics modeling. Despite decades of research, accurately modeling blood flow dynamics across this multi-scale system remains challenging due to its geometric complexity (e.g., asymmetric bifurcations, tortuous pathways) and dynamic heterogeneity in mechanical properties^5^.

Traditional hemodynamic models, such as Hagen-Poiseuille flow in rigid cylindrical tubes^6^ and Womersley’s pulsatile flow theory^7^, laid the foundation for understanding pressure-flow relationships but oversimplified vascular geometry and compliance. Murray’s law^8^, optimizing vessel diameters via energy minimization, advanced the field but relied on symmetric bifurcation assumptions inconsistent with in vivo observations^9^. These idealized frameworks fail to capture the fractal-like branching patterns and heterogeneous scaling behaviors inherent to biological vasculature^10^.

Recent advancements in fractal theories have provided new insights into the characterization of blood flow, particularly through the development of fractal vascular tree models^11,12^. These models offer a promising approach to capture the heterogeneity and complexity of the vascular system, which is essential for understanding cardiovascular diseases^13^. Fractal geometry has long been recognized for its ability to describe the self-similar and scale-invariant properties of natural structures, including the vascular system. Recent studies have demonstrated that fractal models can effectively simulate the branching patterns of blood vessels, which are critical for determining blood flow distribution^14–17^. They can also reflect differences in tissue metabolic requirements. Higher fractal dimensions correspond to denser and more complex branching structures, typically associated with highly vascularized or metabolically demanding tissues^18^. West et al.^19^ pioneered a single-fractal model linking branching patterns to metabolic demands, enabling efficient estimation of global hemodynamic parameters. Chen et al.^20^ established blood flow mathematical models of capillary network and arteriole-capillary fractal network based on fractal theory through theoretical derivation, and analyzed blood flow characteristics, dynamic flow resistance effect and vascular fractal physiological characteristics. Zhou et al.^21^ proposed a fractal operator based on a functional circuit of arterial blood flow, including the shunt effect of branch vessels. However, single-fractal models inadequately address the multi-scale heterogeneity of vascular networks. For instance, microvascular beds exhibit adaptive fractal dimensions modulated by tissue oxygenation^15^, while large arteries follow Murray-like scaling^22^, necessitating multi-fractal frameworks. Recent work demonstrates pronounced spatial variations in fractal dimensions across pathological and physiological contexts, such as tumor vasculature^23^ and cerebral microcirculation^24^, underscoring the need for adaptive, hierarchical models. This concept is reinforced by computational hemodynamics studies showing that flow heterogeneity and transport efficiency are highly sensitive to microvascular structural variability. Multi-scale coronary microcirculation models, for example, demonstrate strong coupling between upstream stenosis, downstream flow distribution, and capillary transit time heterogeneity^25^. Likewise, oxygen-transport simulations reveal that accurately representing microvascular geometric complexity is essential for reproducing physiologically realistic perfusion patterns^26^. These findings collectively underscore that vascular heterogeneity arises across multiple spatial scales, and models must incorporate both macro- and micro-level geometric variability to achieve physiological realism.

The multi-fractal approach extends traditional fractal analysis by considering the variability and non-uniformity of the vascular tree, allowing for a more accurate representation of blood flow dynamics. For example, Van Beek et al.^27^ uncovered the fractality of the relative dispersion of blood flow distribution, while Grasman et al.^28^ described that the distribution of blood flow at the same generation is multifractal. These studies highlight the potential of fractal and multi-fractal methods in characterizing blood flow in the cardiovascular system. Several recent studies have focused on the development and validation of multi-fractal vascular tree models. For instance, Liu et al.^29^ reviewed state-of-the-art computational models of the circle of Willis with physiological applications. Their work emphasizes the importance of multi-scale modeling in understanding blood flow heterogeneity. Similarly, Kharche et al.^30^ conducted a computational assessment of blood flow heterogeneity in peritoneal dialysis patients’ cardiac ventricles, demonstrating the potential of multi-fractal models in clinical applications. Despite these advancements, there are still challenges in fully characterizing blood flow in the cardiovascular system using multi-fractal models.

This study proposes a novel multi-fractal vascular tree (MFVT) model to bridge these gaps. By incorporating fractal dimensions and scaling laws, the MFVT model enables the characterization of blood flow across vascular generations with varying biomechanical properties. Unlike prior approaches, our framework accounts for the interplay between geometric asymmetry, hemodynamic forces, and metabolic demands, thereby offering a more physiologically realistic representation of blood flow distribution. The model integrates three key components: (1) a hierarchical multi-fractal algorithm for generating vascular branching patterns, (2) a hemodynamic solver incorporating Newtonian fluid behavior and vessel compliance, and (3) validation against experimental flow measurements. By quantifying the effects of tortuosity, bifurcation, and scaling laws on flow rate and permeability, the MFVT model provides a unified framework for analyzing multi-scale hemodynamics. This work advances the mechanistic understanding of vascular adaptation and offers a computational tool for studying cardiovascular pathologies.

## Fractal model

**Fig. 1:**
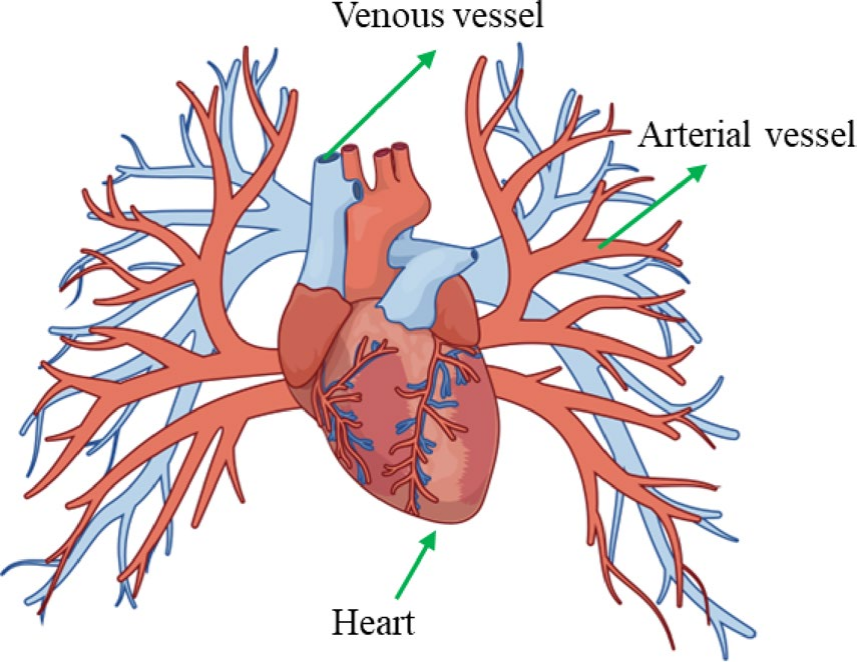
Multistage bifurcated tree vessels in the cardiovascular system.

The vessels in the cardiovascular system have a typical multistage bifurcated tree characteristic, as shown in Fig. 1. This section introduces the MFVT model designed to characterize the blood flow within the cardiovascular system. Initially, the flow rate of blood in an individual vessel, accounting for tortuosity, is established. Subsequently, the flow rate within a fractal vascular tree is derived based on the flow rate of the single vessel. Finally, the flow rate and permeability in a composite structure of fractal vascular trees is determined from the flow rate of the fractal vessel tree. Here, the term “permeability” refers exclusively to the effective hydraulic permeability of the vascular network considered as a porous medium, and does not include the permeability of the surrounding tissue matrix. In this model, the zero-level vessels refer to the largest vessels within each fractal tree hierarchy, serving as the root segments from which all subsequent branches are generated. Their diameters and lengths provide the baseline geometric scale for all lower-level branches through fractal scaling laws. The following assumptions underpin the model derivation: (1) The cardiovascular system is comprised of a network of tortuous fractal vascular tree bundles featuring intersections; (2) The diameters of the blood vessels at the zero-level and the tortuous lengths adhere to fractal scaling laws; (3) The blood within the vessels is treated as Newtonian fluids.

### Blood flow rate in a single vessel

The blood flow rate within an individual vessel is dictated by the generalized Hagen-Poiseuille equation:

1 $$q=\frac{\pi }{{128\mu }}\frac{{\Delta p}}{L}{a^4}$$

where *q* is the blood flow rate, *μ* is the hydrodynamic viscosity, *L* is the vessel length, *a* is the vessel diameter, and *Δ p* is the pressure differential existing between the two endpoints of the vessel.

In practical terms, vessels do not always maintain a straight form; on the contrary, they frequently exhibit a tortuous shape. This tortuous feature has a significant impact on the flow mechanism within the vessels. Assume that ${L_0}$ represents the straight length of vessels and ${L_T}$ represents the tortuous length, the relationship between ${L_0}$ and ${L_T}$ can be depicted by^31,32^:

2 $${L_T}={a^{1 - {D_T}}}L_{0}^{{{D_T}}}$$

where ${D_T}$ is the tortuosity fractal dimension and it can reflect the tortuosity of fluid flow in vessels.

The traditional approach to calculating flow rate in a single tortuous vessel characterized by a fractal distribution of tortuosity involves submitting Eq. (2) into Eq. (1):

3 $$q=\frac{{\pi {a^{3+{D_T}}}\Delta p}}{{128\mu L_{0}^{{{D_T}}}}}$$

It should be noted that directly substituting *L* with *L*_*T*_ implies a prerequisite that the Dean number is less than 40. Otherwise, secondary flows will develop in the channel, causing significant deviation from Poiseuille flow^33^. Departing from the traditional approach, this investigation derived a fresh expression of the flow rate in a single tortuous vessel featuring a fractal distribution of tortuosity based on Newton’s law of viscosity^34^.

**Fig. 2:**
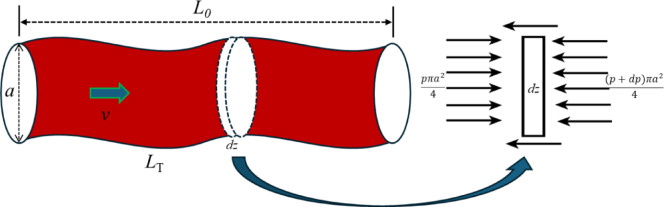
Schematic representation of blood flow in a single tortuous vessel.

Figure 2 illustrates a schematic representation of the blood flow within a tortuous vessel characterized by a fractal distribution of tortuosity. In this context, the blood flow is primarily influenced by viscous forces and the driving force generated by the pressure difference across the two ends of the vessel. Under the assumption that the blood is in a steady state with no acceleration, we can say that the viscous force is balanced by the displacement force, there is:

4 $$\mu \frac{{dv}}{{dr}}(\pi adz)=\left[ {p - (p+dp)} \right]\frac{{\pi {a^2}}}{4}$$

5 $$\mu \frac{{dv}}{{dr}}(\pi a)+\frac{{\Delta P}}{{{L_T}}}\frac{{\pi {a^2}}}{4}=0$$

where *v* is the flow velocity.

When Eq. (2) is submitted into Eq. (5), the differential velocity can be formulated as:

6 $$dv= - \frac{{\Delta P{{(2r)}^{{D_T}}}}}{{4\mu L_{0}^{{{D_T}}}}}dr$$

Next, integrating Eq. (6) will enable us to obtain the blood flow velocity *v*:

7 $$v= - \frac{{{2^{{D_T} - 2}}\Delta P{r^{1+{D_T}}}}}{{\mu L_{0}^{{{D_T}}}(1+{D_T})}}+C$$

where *C* stands the boundary condition. Given that the blood flow velocity on the inner surface of the vessel equals zero, *C* can be ascertained as:

8 $$C=\frac{{\Delta P{a^{1+{D_T}}}}}{{8\mu L_{0}^{{{D_T}}}(1+{D_T})}}$$

By submitting Eq. (8) into Eq. (7), the blood flow velocity *v* at the position *r* can be formulated as:

9 $$v=\frac{{\Delta P({{{a^{1+{D_T}}}} \mathord{\left/ {\vphantom {{{a^{1+{D_T}}}} 8}} \right. \kern-0pt} 8} - {2^{{D_T} - 2}}{r^{1+{D_T}}})}}{{\mu L_{0}^{{{D_T}}}(1+{D_T})}}$$

Thereafter, the blood flow rate *q* within the single fractal tortuous vessel can be acquired from the following equation:

10 $$q=2\pi \int_{0}^{{\frac{a}{2}}} {vrdr}$$

Submitting Eq. (9) into Eq. (10), allows us to derive the expression for the blood flow rate *q* as:

11 $$q=\frac{{\pi \Delta P{a^{3+{D_T}}}}}{{\mu L_{0}^{{{D_T}}}(1+{D_T})}}\left( {\frac{1}{{32}} - \frac{1}{{48+16{D_T}}}} \right)$$

Equation (11) is the new expression of the blood flow rate in a single tortuous vessel with fractal distribution of tortuosity developed in this study. For the case where ${D_T}=1$, Eq. (11) can be simplified as Eq. (1). Therefore, the modified Hagen-Poiseuille equation is a special case of our newly developed model. According to Eq. (11), *q* is directly proportional to *a*^3 + *DT*^, and inversely proportional to ${L}_{0}^{{D}_{T}}$, which is consistent with the findings observed by Wang et al.^25,35^, demonstrating the accuracy of the model.

**Fig. 3:**
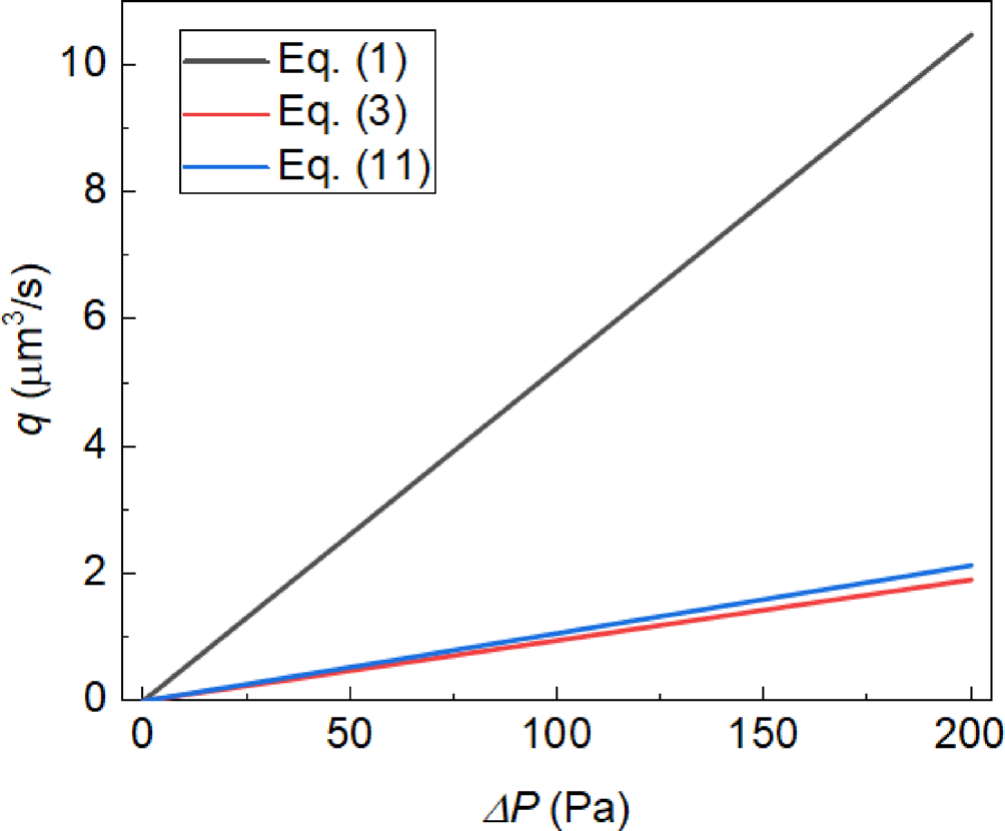
The blood flow rate calculated by different equations for a single vessel with a fractal distribution of tortuosity (*a* = 2 μm, *D*_T_=1.2, *µ* = 0.75 mPa∙s, *L*_0_ = 10 mm).

Figure 3 presents the blood flow rates within a single vessel computed by different equations. When the tortuosity of the vessel is not taken into account, Eq. (1) yields an over - estimation of the blood flow rate. The outcomes of the blood flow rate calculations using Eq. (3) and Eq. (11) are in close agreement. Nevertheless, the blood flow rate determined by the newly developed formula is lower than that derived from the conventional approach. This discrepancy indicates that tortuosity exerts a more pronounced influence on the blood flow within a single vessel featuring a fractal distribution.

### Blood flow rate in a fractal vessel tree

**Fig. 4:**
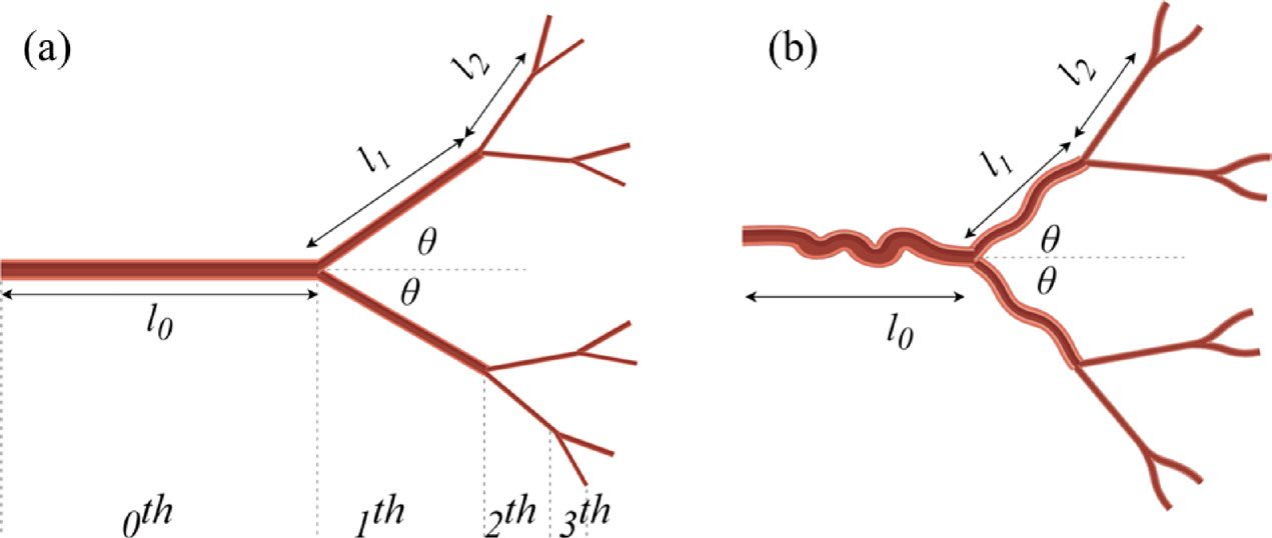
Schematic representation of a fractal-like tree vessel: (**a**) Straight blood vessel, (**b**) Tortuous blood vessel.

In fluid dynamics research, accurately analyzing the blood flow in complex vessels is crucial. As shown in Fig. 4, a single branched vessel can be simplified to a fractal-like tree network. This simplification captures the key branching features and allows us to use fractal-geometry theories. In this section, we systematically derive a mathematical expression for the blood flow rate in a tortuous fractal-like tree vessel network. By integrating fluid-mechanics principles with the network’s geometric properties, this expression helps us understand and predict fluid behavior in such systems^36–38^.

We assume that each branch of the fractal-like tree network consists of a series of self-similar tortuous vessels. In terms of branching, each vessel divides into *n* branches; for a binary tree architecture, *n* = 2. The direction of vessel branching is determined by the branch angle *θ* (0 < *θ* < 90). To facilitate analysis, we assume *θ* remains constant across all branch levels.

The complexity of the fractal-like tree network is quantified by the branch level *m*. For example, *m* = 3 in the networks of Fig. 4(a) and (b). A larger *m* corresponds to a more complex network with an increased number of sub - branches, which significantly impacts blood flow distribution, pressure drop, and system performance^39^. This understanding aids in predicting and optimizing the behavior of such fluid-transport systems.

During each division process, the vessel length and diameter decrease. A typical tortuous vessel at the *k* level (*k* = 0, 1, 2, …, *m*) has length *l*_k_ and diameter *a*_k_. To find the vessel length *l*_k+1_ at the *k* + 1 level, we introduce the length ratio *γ* as

12 $$\gamma ={{{l_{k+1}}} \mathord{\left/ {\vphantom {{{l_{k+1}}} {{l_k}}}} \right. \kern-0pt} {{l_k}}}$$

Likewise, we introduce the diameter ratio *β* to define the relationship between the diameter *a*_k_ of a vessel and that of the vessel at the subsequent level, *a*_k+1_.

13 $$\beta ={{{a_{k+1}}} \mathord{\left/ {\vphantom {{{a_{k+1}}} {{a_k}}}} \right. \kern-0pt} {{a_k}}}$$

In the hierarchical analysis of a vessel network, we assume the zero-level vessel has a defined length *l*_0_ and diameter *a*_0_. These serve as the base parameters for the entire system. Leveraging geometric and physical relationships in the vessel-branching process, we can formulate equations to precisely describe the length and diameter of vessels at the *k* level. The resulting expressions quantify how these properties vary across hierarchical levels, enabling predictions and comparisons within the complex vessel network. Here are the expressions for the length and diameter of the *k* level vessel:

14 $${l_k}={l_0}{\gamma ^k}\:, \:{a_k}={a_0}{\beta ^k}$$

Drawing on the theoretical foundation of Eq. (11), which embodies the physical principles governing blood flow in a hierarchical vessel network, we can formulate an explicit expression for the blood flow rate within a vessel at the *k* level as follows:

15 $${q_k}=\frac{{\pi \Delta {P_k}a_{k}^{{3+{D_T}}}}}{{\mu l_{k}^{{{D_T}}}(1+{D_T})}}\left( {\frac{1}{{32}} - \frac{1}{{48+16{D_T}}}} \right)$$

Consequently, the total flow rate of all *k* level vessels is found by summing their individual blood flow rates:

16 $$q={n^k}{q_k}=\frac{{{n^k}\pi \Delta {P_k}a_{k}^{{3+{D_T}}}}}{{\mu l_{k}^{{{D_T}}}(1+{D_T})}}\left( {\frac{1}{{32}} - \frac{1}{{48+16{D_T}}}} \right)$$

The pressure differential Δ*P*_k_ across the vessel network is:

17 $$\Delta {P_k}=\frac{{\mu l_{k}^{{{D_T}}}{D_T}}}{{{n^k}\pi a_{k}^{{2+{D_T}}}\left( {\frac{1}{{32}} - \frac{1}{{32+16{D_T}}}} \right)}}q$$

Disregarding junction pressure losses, the total Δ*P* between the zero-level vessel and the *m* level vessel is:

18 $$\Delta P=\sum\limits_{{k=0}}^{m} {\Delta {P_k}}$$

By substituting the relationships defined in Eq. (14) and Eq. (17) into Eq. (18), we proceed as follows:

19 $$\Delta P=\frac{{(1+{D_T})\mu l_{0}^{{{D_T}}}}}{{\pi a_{0}^{{3+{D_T}}}\left( {\frac{1}{{32}} - \frac{1}{{48+16{D_T}}}} \right)}}\frac{{1 - {{\left( {\frac{{{\gamma ^{{D_T}}}}}{{n{\beta ^{3+{D_T}}}}}} \right)}^{m+1}}}}{{1 - \frac{{{\gamma ^{{D_T}}}}}{{n{\beta ^{3+{D_T}}}}}}}q$$

Subsequently, the flow rate within a fractal-like tree-structured vessel network can be formulated as follows:

20 $$q=\frac{{\pi a_{0}^{{3+{D_T}}}\left( {\frac{1}{{32}} - \frac{1}{{48+16{D_T}}}} \right)}}{{(1+{D_T})\mu }}\frac{{1 - \frac{{{\gamma ^{{D_T}}}}}{{n{\beta ^{3+{D_T}}}}}}}{{1 - {{\left( {\frac{{{\gamma ^{{D_T}}}}}{{n{\beta ^{3+{D_T}}}}}} \right)}^{m+1}}}}\frac{{\Delta P}}{{l_{0}^{{{D_T}}}}}$$

### Blood flow rate in a bundle of fractal vessel tree

**Fig. 5:**
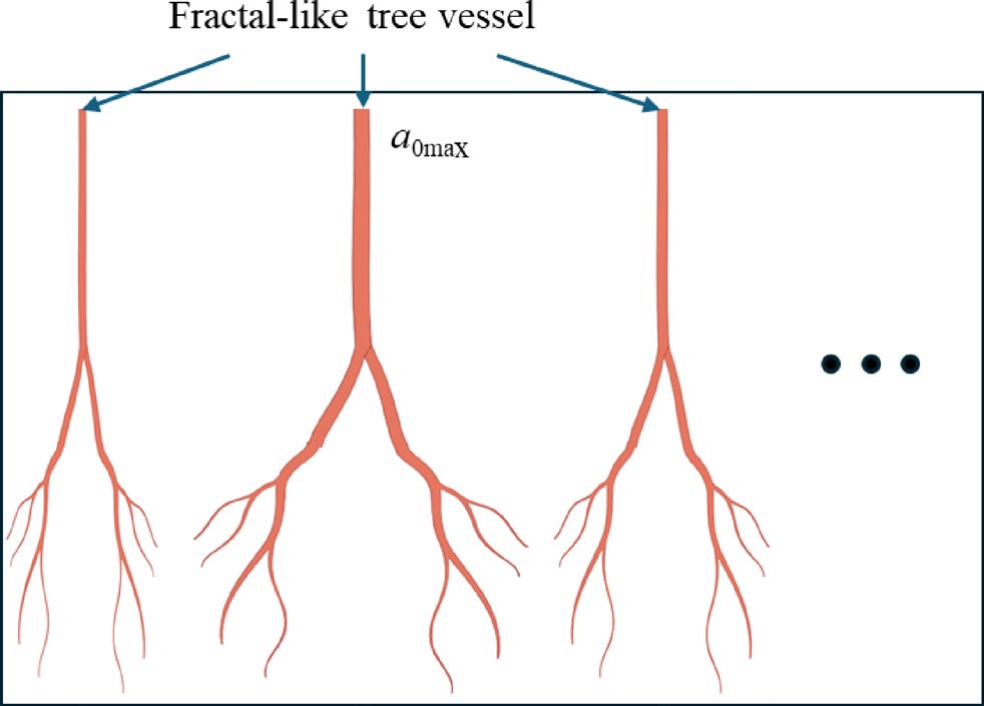
Schematic representation of a bundle of fractal-like tree vessel with different zero-level vessel diameter.

In this study, as illustrated in Fig. 5, we postulate that the vessel system is composed of a bundle of non-intersecting tortuous fractal-like tree vessel networks. We assume that the apertures of the vessels at the zero level adhere to a fractal scaling rule. Based on this assumption, the quantity of fractal - like tree vessel networks can be determined using the following scaling law^40–42^:

21 $$N\left( {a \geqslant {a_0}} \right)={\left( {\frac{{{a_{0\hbox{max} }}}}{{{a_0}}}} \right)^{{D_f}}}$$

where *a*_0max_ is the maximum diameter of the zero-level vessel; *a*_0_ denotes the diameter of the zero-level vessel; *D*_f_ stands for the fractal dimension of the vessel diameter distribution;$N\left( {a \geqslant {a_0}} \right)$ represents the quantity of fractal - like tree vessel networks in which the diameter of the zero-level vessel is larger than *a*_0_.

Under the assumption that Eq. (21) is both continuous and differentiable, the number of fractal-like tree vessel networks for which the diameter of the zero-level vessel lies within the range from *a*_0_ to *a*_0_+*da*_0_ can be formulated as^43,44^:

22 $$- dN\left( {{a_0}} \right)={D_f}a_{{0\hbox{max} }}^{{{D_f}}}a_{0}^{{ - \left( {{D_f}+1} \right)}}d{a_0}$$

The fractal dimension ${D}_{\mathrm{f}}$ of the vessel diameter distribution can be expressed as^45^:

23 $${D_f}=2 - \frac{{\ln \varphi }}{{\ln ({{{a_{0\hbox{min} }}} \mathord{\left/ {\vphantom {{{a_{0\hbox{min} }}} {{a_{0\hbox{max} }}}}} \right. \kern-0pt} {{a_{0\hbox{max} }}}})}}$$

The expression for the tortuosity fractal dimension can be derived as follows^46^:

24 $${D_T}=1+\frac{{\ln {\tau _{{\mathrm{av}}}}}}{{\ln ({{{L_0}} \mathord{\left/ {\vphantom {{{L_0}} {{a_{{\mathrm{av}}}}}}} \right. \kern-0pt} {{a_{{\mathrm{av}}}}}})}}$$

where ${a}_{\mathrm{a}\mathrm{v}}$ is the average diameter of vessels; ${\tau}_{\mathrm{a}\mathrm{v}}$ is the average tortuosity of vessels.

The average diameter of vessels can be precisely determined via the following computational method^47^:

25 $${a_{{\mathrm{av}}}}=\frac{{{D_f}{a_{0\hbox{min} }}}}{{{D_f} - 1}}$$

The average tortuosity of vessels can be computed using the following formula^46^:

26 $${\tau _{{\mathrm{av}}}}=\frac{1}{2}\left[ {1+\frac{1}{2}\sqrt {1 - \varphi } +\frac{{\sqrt {{{(\sqrt {1 - \varphi } - 1)}^2}+\frac{{1 - \varphi }}{4}} }}{{1 - \sqrt {1 - \varphi } }}} \right]$$

The total flow rate of the vessel system can be calculated by aggregating the flow rates of each individual fractal-like tree vessel network:

27 $${Q_v}= - \int_{{{a_{0\hbox{min} }}}}^{{{a_{0\hbox{max} }}}} q dN({a_0})$$

By substituting the expressions presented in Eq. (20) and Eq. (22) into Eq. (27):

28 $${Q_v}=\frac{{\pi {D_f}a_{{0\hbox{max} }}^{{3+{D_T}}}\left( {\frac{1}{{32}} - \frac{1}{{48+16{D_T}}}} \right)}}{{(1+{D_T})\mu \left( {3 - {D_f}+{D_T}} \right)}}\frac{{1 - \frac{{{\gamma ^{{D_T}}}}}{{n{\beta ^{3+{D_T}}}}}}}{{1 - {{\left( {\frac{{{\gamma ^{{D_T}}}}}{{n{\beta ^{3+{D_T}}}}}} \right)}^{m+1}}}}\left[ {1 - {{\left( {\frac{{{a_{0\hbox{min} }}}}{{{a_{0\hbox{max} }}}}} \right)}^{3 - {D_f}+{D_T}}}} \right]\frac{{\Delta p}}{{l_{0}^{{{D_T}}}}}$$

In the tortuous fractal-like tree vessel network model, the equivalent straight length *L* can be derived by summing up the straight lengths of all vessels at each hierarchical level:

29 $$L={l_0}\left( {1+\sum\limits_{{k=1}}^{m} {{\gamma ^k}\cos \theta } } \right)={l_0}\left[ {1+\frac{{\gamma \left( {1 - {\gamma ^m}} \right)}}{{1 - \gamma }}\cos \theta } \right]$$

where *θ* represents the branch angle. Under the assumption that the porosity *φ* remains constant across every cross-section, the diameter ratio can be ascertained through the following method:

30 $$\beta ={{{a_{k+1}}} \mathord{\left/ {\vphantom {{{a_{k+1}}} {{a_k}}}} \right. \kern-0pt} {{a_k}}}={1 \mathord{\left/ {\vphantom {1 n}} \right. \kern-0pt} n}$$

A representative circular cross-sectional area *A* is selected. Subsequently, the vessel area *A*_p_ can be determined as follows:

31 $${A_p}= - \int_{{{a_{0\hbox{min} }}}}^{{{a_{0\hbox{max} }}}} {\frac{{\pi a_{0}^{2}}}{4}} dN({a_0})=\frac{{\pi {D_f}a_{{0\hbox{max} }}^{2}}}{{8 - 4{D_f}}}\left[ {1 - {{\left( {\frac{{{a_{0\hbox{min} }}}}{{{a_{0\hbox{max} }}}}} \right)}^{2 - {D_f}}}} \right]$$

The cross-sectional area *A* can be given by:

32 $$A=\frac{{{A_p}}}{\varphi }=\frac{{\pi {D_f}a_{{0\hbox{max} }}^{2}}}{{\varphi \left( {8 - 4{D_f}} \right)}}\left[ {1 - {{\left( {\frac{{{a_{0\hbox{min} }}}}{{{a_{0\hbox{max} }}}}} \right)}^{2 - {D_f}}}} \right]$$

In accordance with Darcy’s law, the absolute permeability *K*_*v*_ can be computed using the following approach:

33 $${K_v}=\frac{{{Q_v}\mu L}}{{A\Delta p}}$$

By substituting the relationships established in Eq. (28), Eq. (29), and Eq. (32) into Eq. (33), the absolute permeability of the fractal vessel system can be derived as follows:

34 $${K_v}=\frac{{\varphi \left( {2 - {D_f}} \right)a_{{0\hbox{max} }}^{{{D_T}+1}}\left( {\frac{1}{8} - \frac{1}{{12+4{D_T}}}} \right)}}{{(1+{D_T})\left( {3 - {D_f}+{D_T}} \right)l_{0}^{{{D_T} - 1}}}}\frac{{1 - \frac{{{\gamma ^{{D_T}}}}}{{n{\beta ^{3+{D_T}}}}}}}{{1 - {{\left( {\frac{{{\gamma ^{{D_T}}}}}{{n{\beta ^{3+{D_T}}}}}} \right)}^{m+1}}}}\frac{{\left[ {1 - {{\left( {\frac{{{a_{0\hbox{min} }}}}{{{a_{0\hbox{max} }}}}} \right)}^{3 - {D_f}+{D_T}}}} \right]}}{{\left[ {1 - {{\left( {\frac{{{a_{0\hbox{min} }}}}{{{a_{0\hbox{max} }}}}} \right)}^{2 - {D_f}}}} \right]}}\left[ {1+\frac{{\gamma \left( {1 - {\gamma ^m}} \right)}}{{1 - \gamma }}\cos \theta } \right]$$

## Model validation

To verify the accuracy of the model, we compared predicted cardiac coronary blood flow rates against published measurements from four male healthy volunteers (56 years old) obtained from the Bethune First Hospital of Jilin University^20^. Table 1 presents the key physical and physiological parameters of the cardiovascular network system for the subjects involved in the study.

**Table 1:** Parameters of the fractal-like tree vascular network used for model validation.

Case	${a_{0\hbox{min} }}$µm	${a_{0\hbox{max} }}$mm	l_0_ mm	*γ*	*β*	*μ* mPa*·*s	*Δ p* mmHg)	m	*n*	φ
1	10	2	111	0.65	0.79	0.5	111	50	2	0.1
2	10	2	114	0.74	0.79	0.5	107	50	2	0.1
3	10	2	117	0.71	0.79	0.5	117	50	2	0.1
4	10	2	118	0.68	0.79	0.5	104	50	2	0.1

**Fig. 6:**
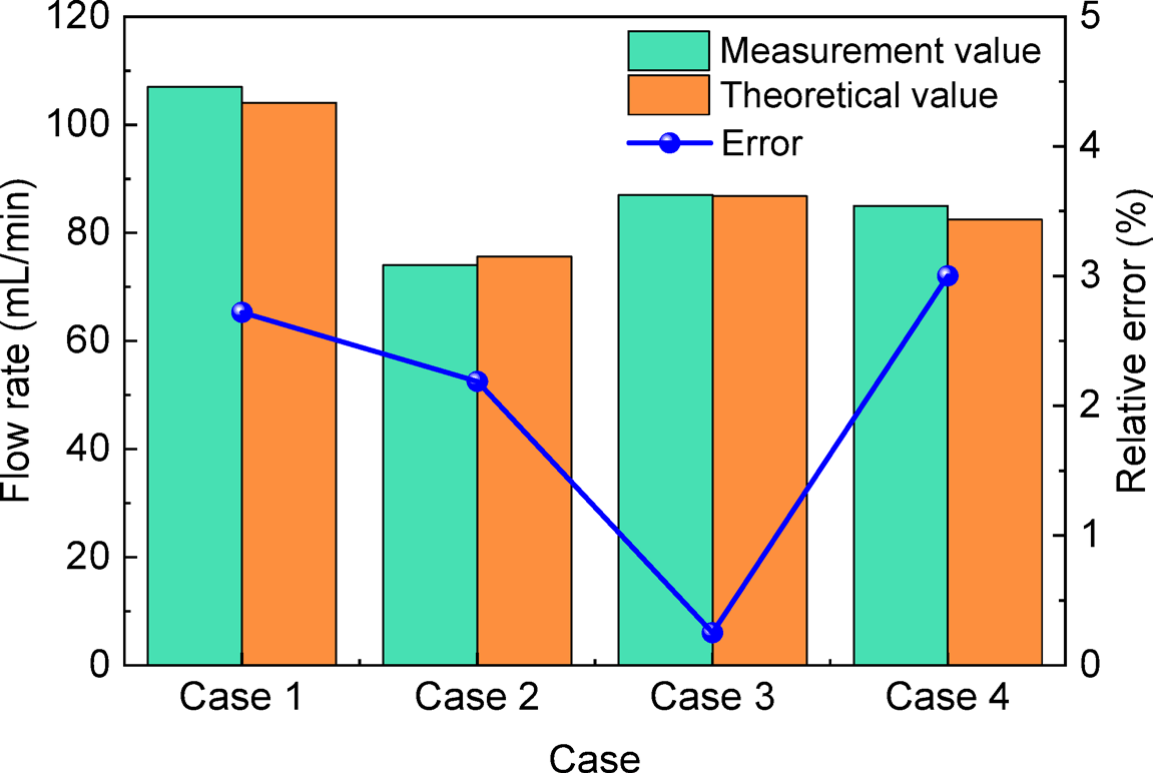
Comparison of the blood flow rate between experimental measured value and theoretical value.

Figure 6 compares the measured and theoretical blood flow rates across four distinct cases, along with the corresponding relative errors. The proposed model demonstrates good overall agreement with the experimental data. Notably, in Cases 2, 3, and 4, the relative errors are low (2.19%, 0.25%, and ~ 3%, respectively), indicating robust predictive performance under varying conditions.

We note that in Case 1, the relative error is 2.72% (theory: 104 mL/min; experiment: 107 mL/min). While this represents a slight increase compared to the other cases, it is crucial to emphasize that the parameters in our model, particularly the tortuosity index, were derived from independent morphological measurements and were not optimized to fit this specific flow rate. This choice prioritizes the model’s physiological basis and generalizability over achieving minimal error for a single output. The marginally higher error in Case 1 may reflect the inherent trade-off when incorporating an additional key anatomical parameter (tortuosity) into a more complex model structure. Importantly, the model’s performance across the wider case set validates its core formulation.

Future work will seek to further refine the estimation of tortuosity from imaging data to enhance the model’s predictive precision without compromising its physiological realism.

## Result analysis

In this research, the proposed MFVT model incorporates a multitude of factors that influence the cardiovascular system’s dynamics. Therefore, in this section, the influences of blood vessel diameter, length, bifurcation, branch level, and volume ratio on the blood flow properties in cardiovascular system will be investigated.

**Fig. 7:**
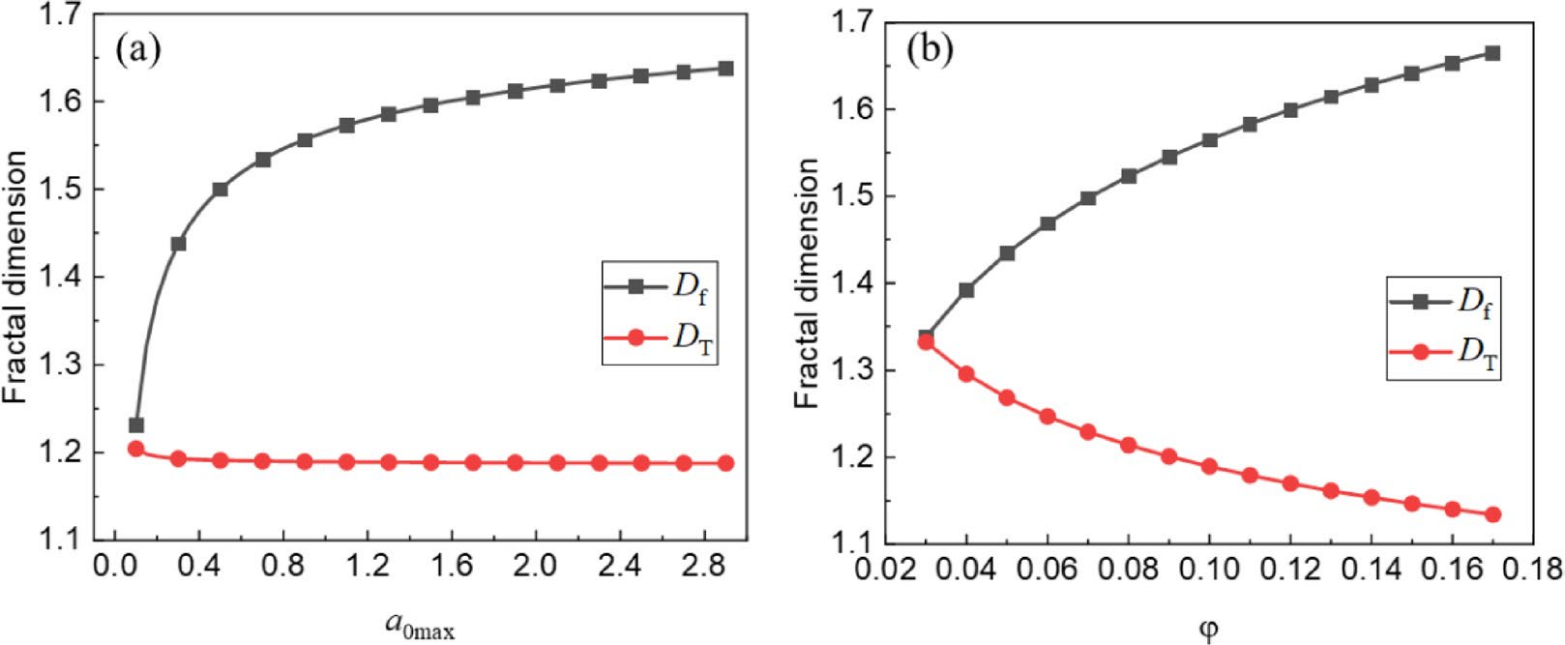
Influence of the maximum diameter of zero-level vessels (**a**) and blood vessel volume ratio (**b**) on fractal dimensions.

The fractal dimension of blood vessels is closely related to the maximum diameter of zero-level vessels and blood vessel volume ratio. Figure 7 depicts the variation of fractal dimensions (*D*_f_ and *D*_T_) with respect to different parameters. In Fig. 7(a), as *a*_0max_ increases from 0 to approximately 2.8, *D*_f_ (represented by black squares) shows a rapid initial rise, particularly noticeable from 0 to around 0.4, followed by a more gradual increase. Conversely, *D*_T_ (in red circles) remains relatively stable with minor fluctuations across the range of *a*_0max_, indicating that *a*_0max_ has a significant impact on *D*_f_ but little effect on *D*_T_.

In Fig. 7(b), with the increase of *ϕ* from 0 to 0.18, *D*_f_ steadily increases, suggesting a positive correlation between *ϕ* and *D*_f_. Meanwhile, *D*_T_ decreases gradually, demonstrating an inverse relationship between *ϕ* and *D*_T_. These findings underscore the distinct influences of *a*_0max_ and *ϕ* on the fractal-dimensional characteristics of the studied system, providing essential insights for further understanding its structural and physical properties.

The physical and physiological parameters of cardiovascular system required for the subsequent parameter sensitivity analysis are shown in Table 2.

**Table 2:** Parameters of the fractal-like tree vascular network used for the model parameter analysis.

Parameter	Benchmark value	Change range
${a_{0\hbox{min} }}$(µm)	10	-
${a_{0\hbox{max} }}$(mm)	2	1–5
*l*_0_ (mm)	10	5–100
*γ*	0.5	0.4–0.6
*β*	0.5	0.5–0.9
*m*	15	5–19
*n*	5	2–10
*θ*	45°	10°−90°
*φ*	0.1	0.05–0.2

### Blood vessel diameter effect

**Fig. 8:**
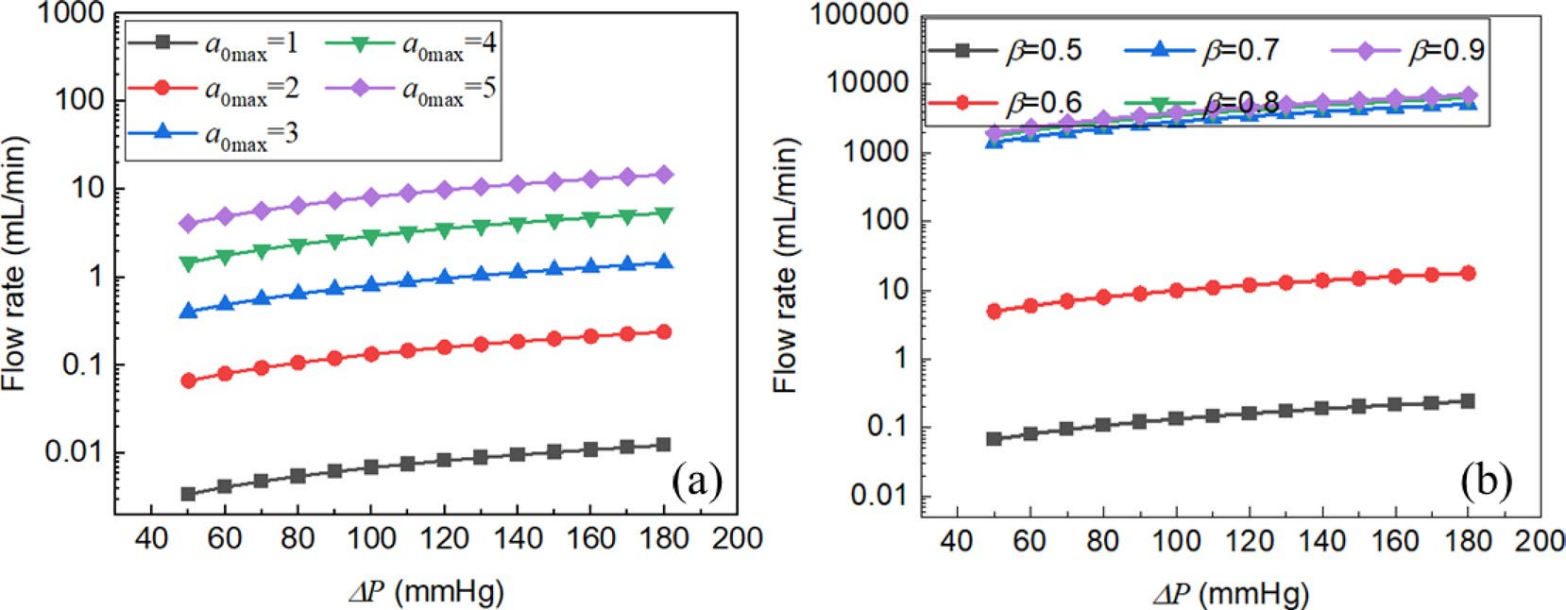
Influence of the maximum diameter of zero-level vessels and vessel diameter ratio on the blood flow rate.

Blood vessel diameter is a significant factor in the progression of cardiovascular diseases. It is associated with a reduction in the production of vasodilatory factors and an increase in the production of vasoconstrictive factors. These changes lead to the proliferation and migration of vascular smooth muscle cells, promoting the thickening and hardening of the vascular walls^48^. Moreover, blood vessel diameter also correlates with the selection of cardiovascular disease treatments. For instance, a study showed that for patients with newly diagnosed, uncomplicated coronary artery disease, the approach of drug-coated balloon (DCB) angioplasty plus bailout stenting did not achieve non-inferiority to planned drug-eluting stent (DES) implantation within two years, suggesting that DES should continue to be the preferred treatment for such patients^49–51^. This indicates that blood vessel diameter is an important factor to consider when selecting treatment methods. The maximum diameter of zero-level vessels (*a*_0max_) and the diameter ratio (*β*) are two such crucial parameters. They directly affect the cross-sectional area available for blood flow passage. Figure 8 shows their impacts on the blood flow rate. Zero-level vessel diameter has been restricted to the physiological range of human coronary artery trunks, approximately 2.3–4.8 mm^52^. The diameter ratio *β* ranges from 0.7 to 0.9, which is consistent with Murray’s law. In this study, the ranges of these two parameters were appropriately expanded.

In Fig. 8(a), it is evident that as *a*_0max_ increases from 1 to 5, the blood flow rate shows a remarkable upward trend for the same range of pressure differences. This indicates a strong positive correlation between *a*_0max_ and the blood flow rate, suggesting that larger zero-level vessel diameters facilitate greater blood transport capacity. Figure 8(b) reveals the influence of *β*. As *β* increases from 0.5 to 0.9, the blood flow rate also increases, especially more prominently at higher pressure differences. This implies that *β* is an important determinant of the blood flow rate, with its effect being more pronounced under higher driving forces.

**Fig. 9:**
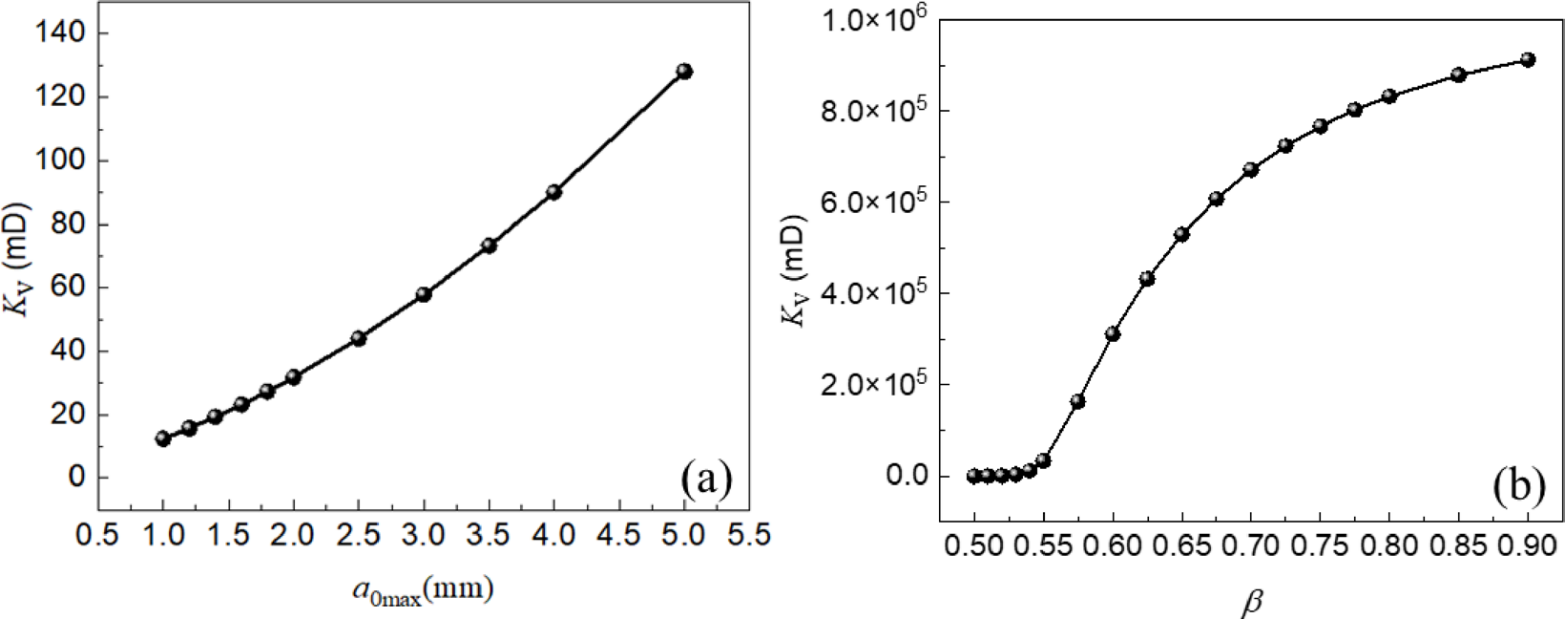
Influence of the maximum diameter of zero-level vessels and vessel diameter ratio on the permeability of the cardiovascular system.

Figure 9 illustrates the relationships between the cardiovascular system permeability and two vascular diameter parameters. In Fig. 9(a), as *a*_0max_ increases from 1 to 5 mm, the cardiovascular system permeability exhibits a nearly linear upward trend. Starting from a relatively low value close to 12.47 mD when *a*_0max_ is minimal, the permeability of the cardiovascular system steadily rises to approximately 128.11 mD at *a*_0max_=5 mm. This indicates a strong positive and relatively consistent correlation between *a*_0max_ and cardiovascular system permeability, suggesting that an increase in the maximum diameter of zero-level vessels directly leads to a proportional increase in the permeability of the cardiovascular system.

In Fig. 9(b), the relationship between cardiovascular system permeability and diameter ratio is more complex. When *β* is in the range of 0.5 to approximately 0.6, the permeability of the cardiovascular system remains at a very low level, close to 0 mD. However, as *β* increases beyond 0.6, the permeability of the cardiovascular system starts to increase rapidly, and as *β* approaches 0.9, the permeability reaches a plateau around 9.0 × 10^5^ mD. This implies that there is a threshold value of *β* (around 0.6) below which *β* has little impact on the permeability of the cardiovascular system, but above which it significantly enhances the cardiovascular system permeability, with the effect gradually diminishing as *β* further increases.

### Blood vessel length effect

The length of blood vessels plays a significant role in the development and treatment of cardiovascular disease. Increased vascular length can be associated with the occurrence and progression of vascular diseases, such as vascular wall inflammation and sclerosis. It is important to note that the relationship between vascular length and cardiovascular disease is complex and can involve various physiological processes. Research has shown that vascular remodeling and the progression of atherosclerosis can be affected by the length of the blood vessels, which in turn influences the choice of treatment methods^53,54^.

**Fig. 10:**
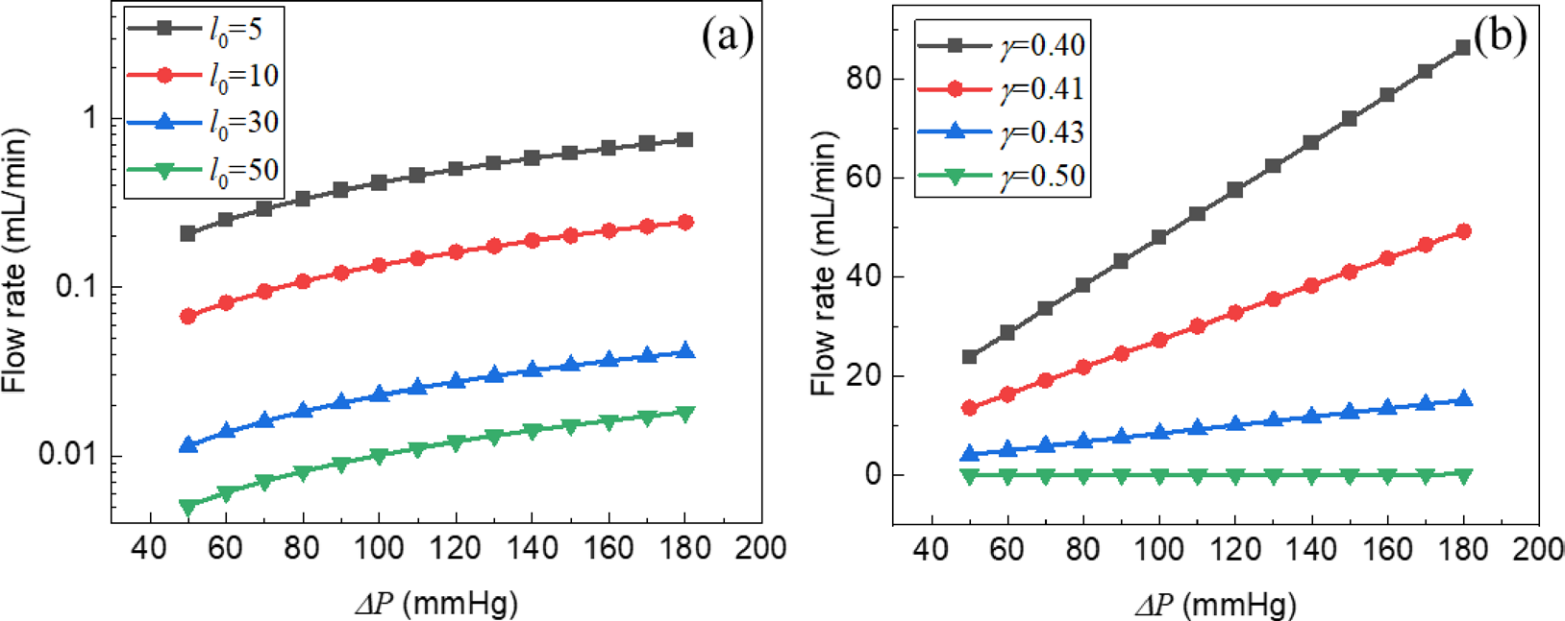
Influence of the length of zero-level vessels and vessel length ratio on the blood flow rate.

In the study of the blood flow phenomena in vascular networks, accurately determining the influencing factors of the blood flow rate is of great significance for understanding physiological and pathological processes. The length of zero-level vessel *l*_0_ and vessel length ratio *γ* are two crucial parameters that may significantly affect the blood flow rate within these networks. Figure 10 presents the influence of these two parameters on the blood flow rate.

In Fig. 10(a), different curves represent the flow rate as a function of the pressure difference for various values of *l*_0_ (*l*_0_ = 5, 10, 30, and 50 mm). It is clear that for a given pressure difference, as *l*_0_ increases, the flow rate decreases. For example, when the pressure difference is 100 mmHg, the flow rate is highest for *l*_0_ = 5 mm and lowest for *l*_0_ = 50 mm. This indicates an inverse relationship between *l*_0_ and the flow rate, suggesting that longer initial lengths may lead to increased resistance and thus reduced flow.

In Fig. 10(b), the curves depict the flow rate - pressure difference relationship for different values of *γ*. Here, as *γ* increases, the flow rate also increases for the same pressure difference. For instance, at the pressure difference of 150 mmHg, the flow rate is lowest for *γ =* 0.4 and highest for *γ =* 0.5. This shows a positive correlation between *γ* and the flow rate, meaning that an increase in *γ* enhances the fluid-carrying capacity of the vascular network under the same pressure-driving force.

**Fig. 11:**
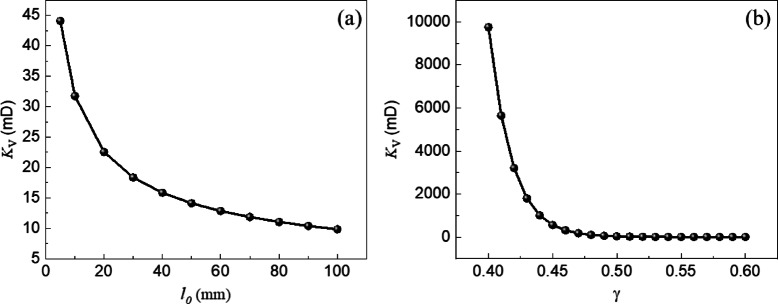
Influence of the maximum length of zero - level vessels and vessel length ratio on the permeability of the vessel system.

Figure 11 displays the relationships between the vascular permeability property and two blood vessel length parameters. As *l*_0_ increases from 0 to 100 mm, the permeability of the vessel system shows a clear downward-trending pattern. Starting at around 45 mD when *l*_0_ is close to 0, the permeability of the vessel system gradually decreases and approaches approximately 10 mD at *l*_0_=100 mm. This indicates an inverse correlation between *l*_0_ and *K*_v_, suggesting that longer initial lengths lead to a reduction in *K*_v_. Such a relationship might be attributed to the increased resistance or altered geometric characteristics of the vascular system as *l*_0_ increases.

With the increase of *γ* from 0.4 to 0.6, the permeability of the vessel system also decreases sharply. Initially, *K*_v_ is around 10,000 mD when *γ =* 0.4, and it drops to a value close to 0 mD as *γ* approaches 0.6. This implies a strong negative correlation between *γ* and *K*_v_. It is likely that changes in *γ* modify certain physical or topological features of the vascular network, which in turn significantly affect *K*_v_.

### Blood vessel bifurcation effect

Vascular bifurcations are critical areas in the cardiovascular system and have a significant relationship with cardiovascular diseases and their treatment. Bifurcations are regions where the flow dynamics are particularly complex due to the split of blood flow from the main vessel into smaller branches, leading to regions of both high and low shear stress^55,56^. This complex flow pattern can contribute to the development of atherosclerosis and plaque formation at these sites, making them more susceptible to disease. Knowledge of how vascular bifurcation affects the blood flow rate can offer valuable insights into physiological functions and may also have implications for pathological conditions related to vascular alterations.

**Fig. 12:**
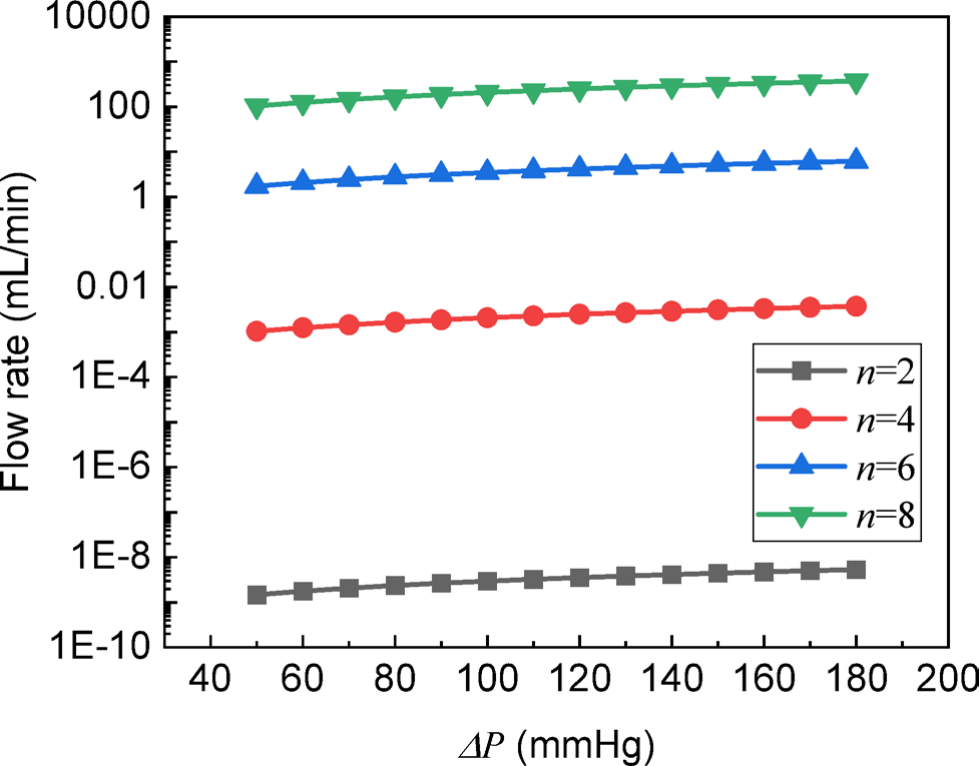
Influence of the vessel branch number on the blood flow rate.

Figure 12 illustrates the relationship between the blood flow rate and the pressure difference for different values of the vessel branch number *n*. It is evident that for a given pressure difference, as the value of *n* increases, the blood flow rate also increases significantly. For example, when the pressure difference is 100 mmHg, the blood flow rate for *n* = 2 is extremely low, in the range of 10^− 8^ − 10^− 6^ mL/min, while for *n* = 8, the blood flow rate is much higher, around 100 mL/min. Additionally, for each value of *n*, the blood flow rate remains relatively stable across the range of pressure difference values considered, showing only minor fluctuations.

**Fig. 13:**
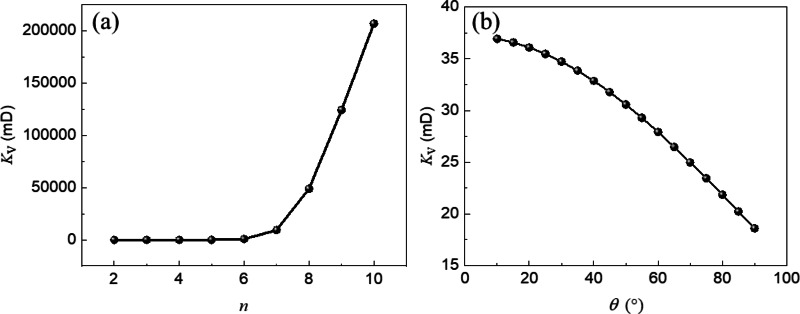
Influence of the vessel branch number and branch angle on the permeability of the vessel system.

Figure 13 presents the relationships between the vascular permeability parameter and two blood vessel bifurcation parameters. As *n* increases from 2 to 10, *K*_v_ initially remains at a relatively low and stable level up to approximately *n* = 6. However, beyond *n* = 6, *K*_v_ experiences a rapid and substantial increase, soaring from a value close to 0 mD to approximately 200,000 mD at *n* = 10. This indicates a non-linear relationship between *n* and *K*_v_, with a threshold-like behavior around *n* = 6, suggesting that changes in *n* have a negligible effect on *K*_v_ below this value but a significant enhancing effect above it.

In Fig. 13(b), as *θ* increases from 0 to 100, *K*_v_ shows a continuous and smooth decrease. Starting at around 37 mD when *θ =* 0, *K*_v_ gradually declines to approximately 17 mD at *θ =* 100. This implies a negative linear correlation between *θ* and *K*_v_, indicating that an increase in the angle *θ* leads to a proportional reduction in *K*_v_.

### Blood vessel branch level and volume ratio effect

**Fig. 14:**
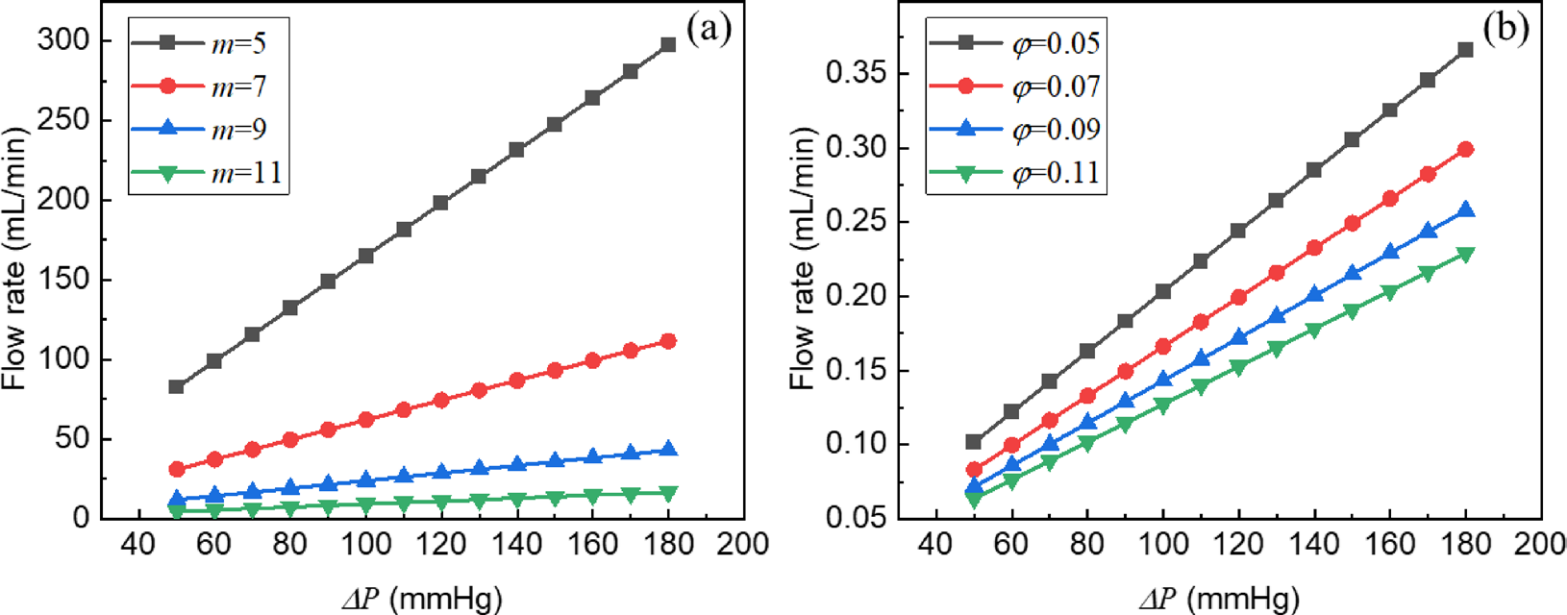
Influence of the blood vessel branch level and volume ratio on the blood flow rate.

The blood vessel branch level influences the distribution of blood flow and oxygen supply to tissues, which can further affect the development and progression of cardiovascular diseases. The hierarchical organization of blood vessels is also important in planning and executing treatments such as angioplasty or bypass surgery, where understanding the branching patterns can help in restoring optimal blood flow to affected areas. Whether it is arteriosclerosis or heart failure, the volume proportion of blood vessels undergoes significant changes. The relationship between the blood flow rate and the pressure difference for different values of the blood vessel branch level *m* and volume proportion of blood vessels is shown in Fig. 14.

The maximum branching level has been limited to *m* = 19, which aligns with reported arterial-tree depths for regional vascular networks^57^. The blood flow rate increases as *m* increases. For example, when the pressure difference is 100 mmHg, the blood flow rate for *m* = 5 is approximately 100 mL/min, while for *m* = 11, it is significantly higher, around 250 mL/min. Additionally, the flow rate - pressure difference relationships for all *m* values are approximately linear, indicating a direct proportionality between blood flow rate and pressure difference for each specific *m*.

Similar to the case of *m*, the blood flow rate increases with an increase in *ϕ*. For instance, at the pressure difference of 150 mmHg, the flow rate for *ϕ=0.05* is around 0.2 mL/min, while for *ϕ=0.11* it is approximately 0.3 mL/min. The blood flow rate - pressure difference relationships for different *ϕ* values also show a linear trend, suggesting a direct and proportional relationship between blood flow rate and pressure difference for each *ϕ*.

**Fig. 15:**
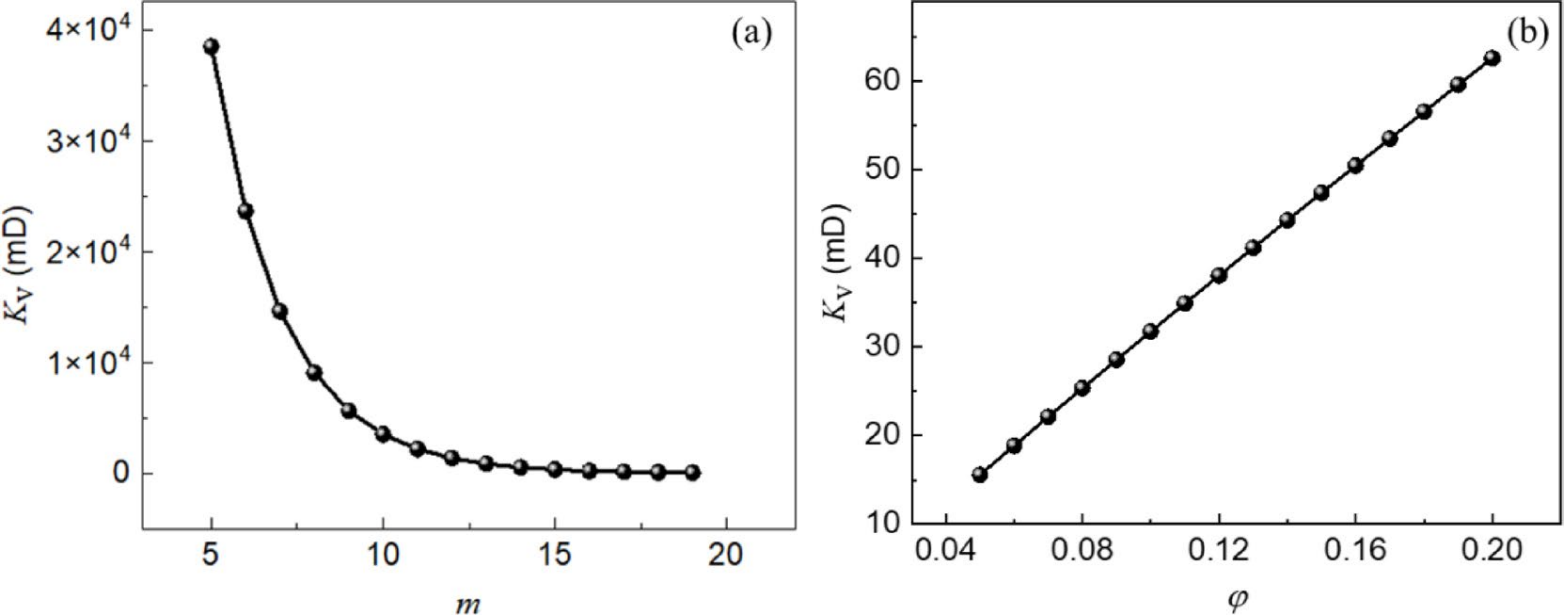
Influence of the blood vessel branch level and volume ratio on the permeability of the vessel system.

Figure 15 illustrates the relationships between the vascular permeability property and blood vessel branch level and volume ratio. As *m* increases from 5 to 19, *K*_v_ exhibits a steep decline. Starting at approximately 4 × 10^5^ mD when *m* = 5, *K*_v_ rapidly decreases and approaches a value close to 50.84 mD as *m* reaches 19. This indicates a strong inverse correlation between *m* and *K*_v_, suggesting that an increase in *m* leads to a significant reduction in the value of *K*_v_. Such a relationship might be attributed to changes in the structural or hydraulic characteristics of the vascular system associated with variations in *m*.

With the increase of *ϕ* from 0.04 to 0.20, *K*_v_ shows a clear upward-trending pattern. Commencing at around 10 mD when *ϕ=0.04*, *K*_v_ steadily rises to approximately 60 mD at *ϕ=0.20*. This implies a positive linear correlation between *ϕ* and *K*_v_, meaning that an increase in *ϕ* is accompanied by a proportional increase in *K*_v_. It is likely that *ϕ* influences certain physical properties of the vascular network, such as porosity or permeability, which in turn affect *K*_v_.

## Discussions

The present study introduces a novel MFVT model to characterize hemodynamic behavior in the cardiovascular system, addressing critical gaps in existing models by incorporating fractal geometry, tortuosity, and hierarchical branching dynamics. Our findings demonstrate that the MFVT framework provides a robust mechanistic understanding of blood flow distribution across vascular generations, validated by experimental data with a maximum relative error of < 5%. This section contextualizes the key results, discusses their implications, and identifies limitations and future directions.

### Key findings and model advancements

The MFVT model successfully integrates heterogeneous fractal dimensions and scaling laws to resolve longstanding challenges in modeling multi-scale hemodynamics. Unlike traditional Hagen-Poiseuille or Murray’s law-based approaches, which oversimplify vascular geometry and bifurcation symmetry^58,59^. Our framework accounts for tortuosity-induced flow resistance (Fig. 3) and adaptive fractal branching patterns. The derived permeability expression (Eq. 34) explicitly links vascular architecture to system-level hemodynamic performance, revealing that permeability increases exponentially with zero-level vessel diameter but declines sharply with branch level (Figs. 9 and 15). These results align with clinical observations of reduced microvascular perfusion in hypertension^9^ and elevated large-artery flow in aneurysms^60^, suggesting that MFVT captures physiologically relevant scaling behaviors. The sensitivity analyses further highlight diameter ratio and branch number as dominant regulators of flow rate (Figs. 8b and 12), corroborating in vivo studies on bifurcation optimization in coronary networks^61^.

### Comparison with prior models

While single-fractal models^58,62^ provided foundational insights into metabolic scaling, they inadequately address the hemodynamic heterogeneity observed in multi-scale vascular networks. The MFVT model advances these works by incorporating spatially varying fractal dimensions (*D*_f_, *D*_T_), which reflect anatomical variations from elastic arteries to tortuous capillaries. This aligns with imaging studies showing region-specific fractal dimensions in cerebral microcirculation^63^ and tumor vasculature^9^. Furthermore, our tortuosity-corrected flow rate equation (Eq. 11) resolves the overestimation bias of classical Hagen-Poiseuille models (Fig. 3), demonstrating that tortuosity reduces flow rates by increasing the effective hydraulic path length. This result differs from the reduced resistance reported by Wang and Bassingthwaighte for small curved tubes^64^, and the reason lies in the fundamentally different hemodynamic regimes addressed by the two models. Their study focused on microvessels in which blood exhibits strong non-Newtonian two-phase behavior dominated by the Fahraeus-Lindqvist effect, where curvature induces lateral migration of erythrocytes and may lower hydraulic resistance. In contrast, the MFVT model applies to relatively large blood vessels, where blood behaves as a homogeneous Newtonian fluid with nearly constant apparent viscosity^65^; in this regime, tortuosity primarily increases the effective path length and thus increases resistance. Our model addresses the limitations of assuming straight vessels in macroscale hemodynamic simulations and emphasizes the need for geometric realism in pathological models, such as atherosclerotic stenosis, where tortuosity is exacerbated^66^.

### Clinical and pathophysiological implications

The parameter sensitivity results offer actionable insights for cardiovascular diagnostics and interventions. The strong dependence of flow rate on *a*_0max_ (Fig. 8a) and *β* (Fig. 8b) suggests that therapies targeting vascular remodeling, e.g., angiotensin inhibitors to reduce arterial stiffening^67^ or DCB angioplasty to optimize lumen dimensions^68^ could be personalized using MFVT-derived permeability metrics. Similarly, the inverse relationship between branch angle (*θ*) and permeability (Fig. 13b) may explain why bifurcation lesions exhibit higher restenosis rates post-stenting^69^, guiding device design to minimize flow disruption. For microvascular pathologies like hypertension, the model predicts that increased vessel length (*l*_0_) and volume ratio (*φ*) reduce perfusion (Figs. 10a and 14b), consistent with capillary rarefaction observed in end-organ damage^70^.

### Limitations and future directions

While the MFVT model represents a significant advance, several limitations warrant consideration. First, blood was modeled as a Newtonian fluid, neglecting shear-thinning behavior and erythrocyte aggregation effects in microvessels. As demonstrated by Pries et al.^63^, blood viscosity remains nearly constant only when vessel diameters exceed a certain size. Therefore, our model is applicable only to relatively large blood vessels. Future iterations could incorporate Casson or Carreau-Yasuda constitutive equations to improve small-vessel accuracy^27^. Second, the assumption of fixed fractal dimensions across generations may not fully capture adaptive vascular remodeling in chronic diseases. Coupling MFVT with oxygen-dependent fractal regulation algorithms^9^ could enhance physiological fidelity. Third, experimental validation was limited to coronary flow rates; in vivo pressure measurements across vascular generations and imaging-based tortuosity quantification are needed for comprehensive validation. Forth, extending the model to simulate pulsatile flow and wall compliance would enable applications in aneurysm rupture risk assessment and ventricular-vascular coupling analysis. Finally, the model assumes that the system can be represented as a set of non-intersecting tortuous fractal vascular trees. This idealized structural assumption simplifies the analysis but also introduces important constraints. Real vascular networks frequently contain anastomoses and collateral pathways that enable pressure equilibration, bypass flow, and redistribution of perfusion between adjacent territories. Because these connections are not included in the current formulation, the model may not accurately represent hemodynamics in regions with dense anastomotic coupling. The framework is therefore most suitable for vascular domains dominated by hierarchical tree-like branching, whereas its use in tissues characterized by extensive microvascular meshes should be approached with caution.

By unifying fractal geometry, hemodynamic principles, and multi-scale vascular architecture, the MFVT model provides a transformative framework for analyzing cardiovascular function. Its ability to quantify the effects of tortuosity, bifurcation asymmetry, and hierarchical branching on flow distribution addresses critical limitations of conventional models. The parametric insights into diameter, length, and bifurcation effects offer mechanistic explanations for clinical phenomena while guiding therapeutic innovation. Future integration with patient-specific imaging and machine learning-based parameter optimization could position MFVT as a cornerstone tool in precision hemodynamics and cardiovascular drug development.

## Conclusions

In this research, a novel MFVT model was developed to precisely characterize the blood flow characteristics in the cardiovascular system, effectively addressing the long - standing challenge of describing hemodynamics caused by the system’s complex geometry and dynamic heterogeneity.

Regarding model construction, the MFVT model comprehensively considered features such as vessel branching and tortuosity, incorporated heterogeneous fractal dimensions and scaling laws. It derived blood flow rate formulas at different vascular scales and obtained the analytical expression for the permeability of the cardiovascular system, offering a more physiologically realistic theoretical framework for hemodynamic research.

The model validation results demonstrated that through the study of different cases, the maximum relative error between the model calculation results and experimental measurements was less than 5%, indicating the rationality and applicability of the model in simulating the blood circulation of the cardiovascular system.

The sensitivity analysis revealed that multiple parameters significantly affected blood flow characteristics and vascular permeability. The maximum diameter of zero-level vessels and the diameter ratio were positively correlated with the blood flow rate and permeability, but with different influencing mechanisms. The initial length of zero-level vessels was negatively correlated with the blood flow rate and permeability, while the vessel length ratio was positively correlated with the blood flow rate and negatively correlated with permeability. The number of vessel branches, branch angle, branch level, and volume ratio also influenced the blood flow rate and permeability in different patterns.

Overall, the findings of this study provide new theoretical insights for a deeper understanding of the hemodynamic mechanisms in the cardiovascular system. They contribute to further exploring abnormal blood flow related to cardiovascular diseases and offer potential theoretical support for the diagnosis, treatment, and drug development of cardiovascular diseases in the future. Future research can further refine the model by considering more physiological factors and expand its applications in clinical practice and biomedical engineering.

## Data Availability

Interested parties can contact the corresponding author to submit a data access request. The corresponding author will review requests and ensure that all data sharing complies with ethical and legal requirements.
